# Morphologically defined sub-stages of *C. elegans* vulval development in the fourth larval stage

**DOI:** 10.1186/s12861-015-0076-7

**Published:** 2015-06-12

**Authors:** Darren Z. L. Mok, Paul W. Sternberg, Takao Inoue

**Affiliations:** Department of Biochemistry, Yong Loo Lin School of Medicine, National University of Singapore, 8 Medical Drive, Blk MD7, #02-06, Singapore, 117597 Singapore; HHMI and Division of Biology and Biological Engineering, California Institute of Technology, Pasadena, CA 91125 USA

**Keywords:** *C. elegans*, Vulval development, Developmental timing, Gene expression

## Abstract

**Background:**

During the fourth larval (L4) stage, vulval cells of *C. elegans* undergo extensive morphogenesis accompanied by changes in gene expression. This phase of vulval development, occurring after the well-studied induction of vulval cells, is not well understood but is potentially a useful context in which to study how a complex temporal sequence of events is regulated during development. However, a system for precisely describing different phases of vulval development in the L4 stage has been lacking.

**Results:**

We defined ten sub-stages of L4 based on morphological criteria as observed using Nomarski microscopy (L4.0 ~ L4.9). Precise timing of each sub-stage at 20 °C was determined. We also re-examined the timing of expression for several gene expression markers, and correlated the sub-stages with the timing of other developmental events in the vulva and the uterus.

**Conclusions:**

This scheme allows the developmental timing of an L4 individual to be determined at approximately one-hour resolution without the need to resort to time course experiments. These well-defined developmental stages will enable more precise description of gene expression and other developmental events.

## Background

For over 30 years, the vulval development of *C. elegans* has been an important model in which to study mechanisms underlying the development of complex organisms [1, 2]. However, most studies of vulval development focused on cell fate specification and inductive interaction during the third larval (L3) stage [3]. The subsequent development of induced vulval cells, during which cell fates determined in the L3 stage are "executed" has been less studied. During the fourth larval (L4) stage, these cells undergo a complex series of morphogenetic events accompanied by dynamic changes in gene expression patterns. This makes it a potentially powerful system in which to study gene regulation during terminal differentiation and mechanisms that underlie complex morphogenetic processes [4–10].

Recent studies of vulval development during the L4 stage follow the detailed description published in 1999 [7]. This work, based on electron microscopy of serial sections and fluorescent labeling of cell-cell junctions, revealed the general sequence of events during the L4 stage. First, vulval cells migrate from where they were generated (near the original positions of vulval precursor cells induced in the L3 stage) toward the center of the future vulva. Second, these cells extend processes and fuse with one another such that they form a dorsal/ventral stack of seven toroids, called vulF, vulE, vulD, vulC, vulB2, vulB1 and vulA. Most of these toroids are syncytial cells with two or four nuclei. The only exceptions are vulB1 and vulB2, which remain unfused, but nevertheless arrange themselves in a ring configuration. Subsequently, the shapes of these cells change further, forming the adult structure that serves as the conduit for developing embryos and for sperm when mating with a male. During this process, additional cell-cell connections are made; vulC and vulD make connections to the vulval muscle cells that open the vulva during egg laying, vulE makes a structural connection to lateral hypodermal cells and vulF makes a connection with uv1 cells of the uterus.

Additional studies led to identification of a number of genes involved in this stage of vulval development and understanding of some morphogenetic processes. Polarized migration of vulval cells requires the signaling protein SMP-1/semaphorin and its receptor PLX-1/plexin, as well as small GTPases MIG-2 and CED-10 (members of the Rho/Rac family) and the GTP/GDP exchange factor UNC-73/Trio [8]. Some of these proteins show polarized localization in each vulval cell. Fusion of vulval cells into syncytial toroids requires fusogens AFF-1 and EFF-1 [11, 12]. The zinc finger transcription factor VAB-23 is a target of regulation by the EGF pathway during the L3 stage, and regulates expression of genes including *smp-1*, thereby linking vulval induction to regulation of morphogenesis in the L4 stage [13]. Finally, morphogenetic movements that shape the developing vulva are a result of complex interplay of various forces operating among the vulval toroids [9]. These forces include contraction of ventral toroids, requiring contraction of actin microfilaments and regulated by the Rho kinase LET-502 [9], as well as generation of dorsal lumen through transient invasion of the anchor cell into the developing vulva [10].

A separate line of investigation looked at genes that are differently expressed in the seven cell types and mutations that affect their expression. Approximately 30 genes are now known to exhibit cell type specific expression among vulval cells in the L4 and/or the adult stage ([14] and references therein). Importantly, each cell type expresses a unique combination of genes, while each gene may be expressed in a single vulval cell type or in multiple cell types. Moreover, the timing of gene expression shows considerable complexity. Expression of different genes in a single cell type can initiate at different time points, and expression of a single gene in different vulval cell types can start at different time points.

The progress in understanding how expression of these genes is controlled has been slow, probably because it is relatively difficult to isolate mutations that affect cell fate or gene expression during the L4 stage. This may be because many of the genes involved in this stage of vulval development are pleiotropic and are required for earlier stages of development [15, 16]. Among the classical lineage mutants studied by Horvitz *et al.*, only *lin-11* appears to have a phenotype consistent with cell fate change at this stage [17, 1]. Additional genes (e.g. *lin-29, egl-38, cog-1, bed-3, nhr-67, vab-23*) were isolated from other screens [18–22, 13]. However, many more genes are likely to regulate this stage of vulval development given the complexity of this system.

Among the known genes regulating gene expression in the L4 stage vulva, a subset demonstrates a possible connection to the heterochronic pathway regulating stage-specific gene expression. In particular, *lin-29* (encoding a zinc-finger transcription factor) is a well-known heterochronic gene regulating the L4-to-adult transition [23]. Moreover, *bed-3* (encoding a BED-type zinc-finger transcription factor) was recently discovered to be regulated by *blmp-1,* another component of the heterochronic pathway [24] (our results not shown). These results suggest that the timing mechanism operating throughout the entire body of the worm feeds into vulval development at specific time points, allowing for precise temporal control of gene expression. However, details of how the temporal sequence of events is regulated within the L4 stage, and how the heterochronic pathway regulates this sequence, are unclear.

In relation to these possibilities, one limitation of previous analyses of L4 development has been the lack of precise timing information. In various contexts, L4 stage animals were classified as "early", "middle" and "late" without a precise definition of each phase (e.g. [4, 5]). In order to fully understand vulval development in the L4 stage, further studies must rely on improved description of developmental timing. Here, we present a further subdivision of the L4 stage into sub-stages (L4.0 to L4.9) based on morphological criteria in the vulva as observed by Nomarski differential interference contrast microscopy. This scheme allows staging of an L4 animal at approximately one hour resolution without the need to follow an individual animal over the course of its development. We correlate our sub-stage scheme with developmental timing when the worms are grown at 20 °C. We also present improved measurement of gene expression timing for several well-characterized vulva-expressed genes.

## Results

### Description of L4 sub-stages defined by morphological characteristics of the vulva

**Fig. 1:**
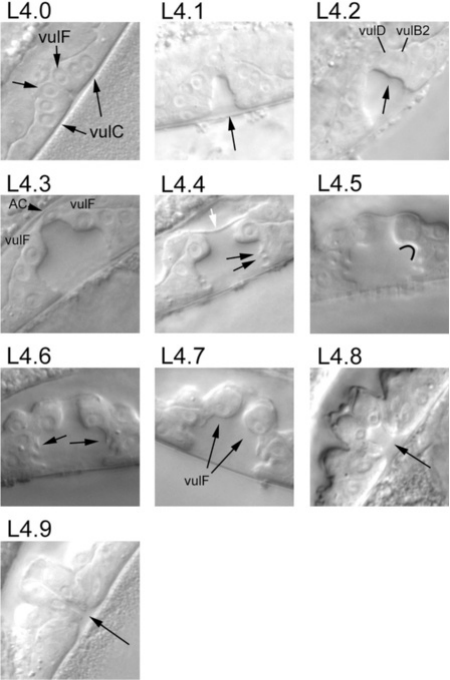
Nomarski images of *C. elegans* vulva at different L4 sub-stages (L4.0 – L4.9). Each sub-stage is distinguished from another by the morphological appearance of the vulva (Table 1). All images in this figure were taken in the focal plane corresponding to the midline of the worm. L4.0: arrows point to undivided cells. L4.1: arrow points to the vulval lumen. L4.2: arrow points to the "kink" between vulC and vulD cells. vulCs are out of the plane of focus. L4.3: the anchor cell and vulF cells are labeled. L4.4: the utse cell separating the vulva lumen from the uterine lumen is indicated by the white arrow. Black arrows point to "fingers" which form on sides of the vulva. L4.5: the concave curve between vulD and vulB2 is shown (black arc). L4.6: ventrally oriented "fingers" are indicated by black arrows. L4.7: the opening between vulF cells are starting to narrow. L4.8, L4.9: the vulval lumen (black arrow) collapses during these stages

To facilitate classification of L4 animals into different developmental sub-stages, we selected morphological criteria that are observed only at or after a particular point during development. The entire set of criteria selected and examples of Nomarski images are shown in Table 1 and Fig. 1. For example, in the early L4 stage, the apex of the vulval lumen lies within the vulval tissue and is pointed at the dorsal end. However, later in development, the lumen extends to the top of the vulval cell layer and the dorsal end becomes flattened.

**Table 1:** Sub-stages of L4

Sub-stage	Description	Approximate time from middle of the L3-to-L4 lethargus	n*
L4.0	vulA, vulB and vulE have divided, but vulC and vulF have not.	0.3	11
L4.1	vulC and vulF have divided and a narrow lumen has formed.	1.1	13
L4.2	Lumen has widened and a prominent kink has formed between vulC and vulD.	2.8	31
L4.3	vulFs have separated and the apex of the lumen is flat and capped by the anchor cell.	4.1	21
L4.4	utse is visible as a thin layer separating the vulval and uterine lumens. “Fingers” are formed at the sides of the vulva next to vulB1 and vulB2.	5.0	26
L4.5	Side of the vulval lumen between vulC and vulD forms a smooth curve.	6.4	45
L4.6	“Fingers” between vulB2 and vulC are pointed ventrally.	7.5	23
L4.7	vulFs have migrated closer such that the lumen is narrowed in the dorsal section. The approximate cutoff is when the width of the channel is less than the width of the vulD nucleus.	8.3	9
L4.8	Lumen is partially collapsed.	8.5	4
L4.9	Lumen is completely collapsed.	8.8	5
Adult		9.7	9

*Number of animals scored at each stage

**Fig. 2:**
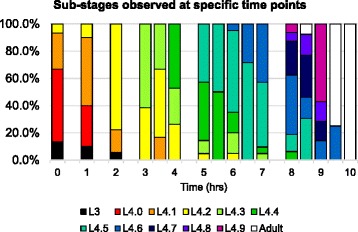
Sub-stages of wild-type worms at specific time points after the L3-to-L4 lethargus. Sub-stages were determined every hour and also at 3.5, 5.5, 6.5, 8.5 and 9.5 h after synchronization. At least ten animals were observed every hour up to eight hours, and at least four animals were observed at all other time points

**Fig. 3:**
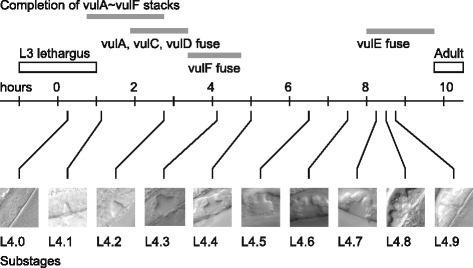
Timeline of sub-stages and developmental events in the L4 stage

To determine the actual timing of each sub-stage, we synchronized populations of wild-type *C. elegans* at the L3-to-L4 lethargus. These populations were then allowed to grow for specific durations at 20 °C and staged according to Table 1 (Methods, Fig. 2). At each time point, two to several stages were observed simultaneously. This probably reflects the fact that the L3-to-L4 lethargus is about two hours long at 20 °C [25, 26]. To determine the approximate timing of each sub-stage, we took the average time point for all observations of each L4 sub-stage (Table 1, Fig. 3). We expect that this would correspond to the approximate time taken from the middle of the L3-to-L4 lethargus to the middle of a given sub-stage. We found that most of the sub-stages were spaced apart by approximately one to two hours.

### Precise timing of reporter gene expression in the L4 stage

**Fig. 4:**
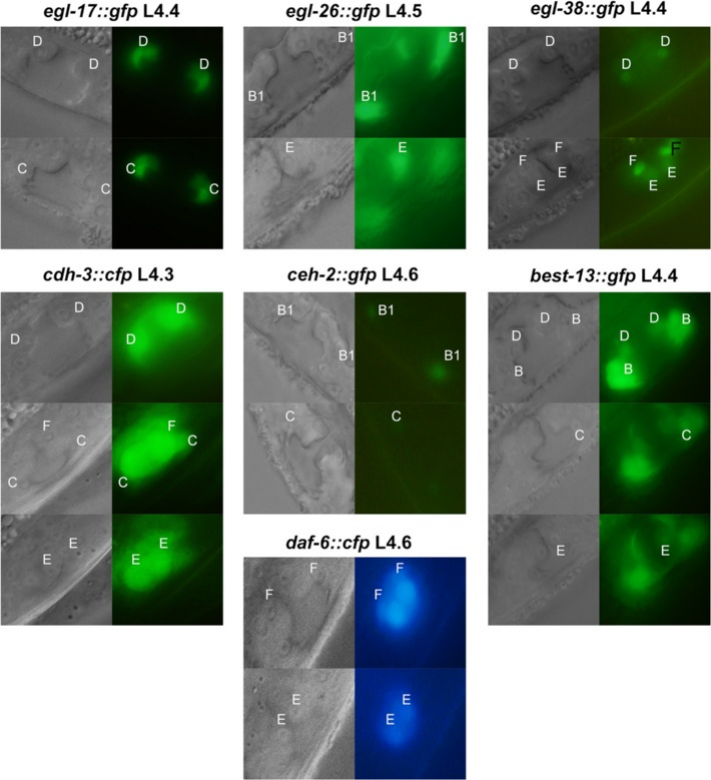
Expression of reporters in sub-stages. Each set of images shows the Nomarski image to the left and the corresponding fluorescence image to the right. The location and identity of vulval cells are indicated by "A" through "F"

The expression pattern of a number of genes in the L4 stage vulva has been reported (e.g. [4]). However, most of these reported expression patterns lack precise timing information. We therefore re-examined expression of ten reporters (Table 2, Fig. 4, Methods). Our results were largely consistent with approximate timing reported previously. "Early-L4" roughly corresponds to sub-stages L4.0 to L4.3, "mid-L4" roughly corresponds to sub-stages L4.4 to L4.6 and "late-L4" roughly corresponds to sub-stages L4.7 to L4.9. Some researchers have used the term "Christmas tree stage" to identify mid-L4 animals with specific morphological features (e.g. [27]). In our scheme, this corresponds to sub-stages L4.4, L4.5 and L4.6.

**Table 2:** Duration of expression for vulval gene expression markers

Reporter	Cells	Duration of GFP expression	Comments
*egl-17::gfp*	C, D	L4.2 to L4.9*	[34]
*cdh-3::cfp*	C, D, E, F	L4.1 to L4.9*	[35] Variable expression in L4.1; consistent in later sub-stages
*ceh-2::gfp*	B1, B2, C	L4.1 to L4.9*	[4] Variable expression in all sub-stages; low level of expression in B2, C from L4.1 to L4.7
*daf-6::cfp*	E, F	L4.3 to L4.9*	[36] Expression in E variable in L4.3 and L4.4, consistent in later sub-stages; expression in F variable throughout
*B0034.1::gfp*	E, F	No expression in L4	[4] Expressed in adults only
*F47B8.6::gfp*	C, D, E, F	No expression in L4	[4] Expressed in adults only
*egl-26::gfp*	B1, B2	L4.4 to L4.9*	[32]
	E	L4.2 to L4.9*	
*egl-38::gfp*	D, E, F	L4.1 to L4.7	[22] Only reporter in this study where expression was obviously turned off during L4.
*best-13::gfp*	B1, B2, C, D, E	L4.1 to L4.9*	[37] Variable expression possibly due to loss of extrachromosomal array

*Expression of these reporters persist into the adult stage

Interestingly, expression of *cdh-3::cfp*, reported to begin in early-L4, and expression of *ceh-2::gfp*, reported to begin in mid-L4 [4], actually started at the same sub-stage L4.1. Although differences in assay conditions could account for the discrepancy, this is perhaps more likely due to differences in the definition of "early-L4" and "mid-L4" used in different experiments, highlighting the need for better staging of L4 animals.

### Correlation of L4 sub-stages with other developmental events

**Fig. 5:**
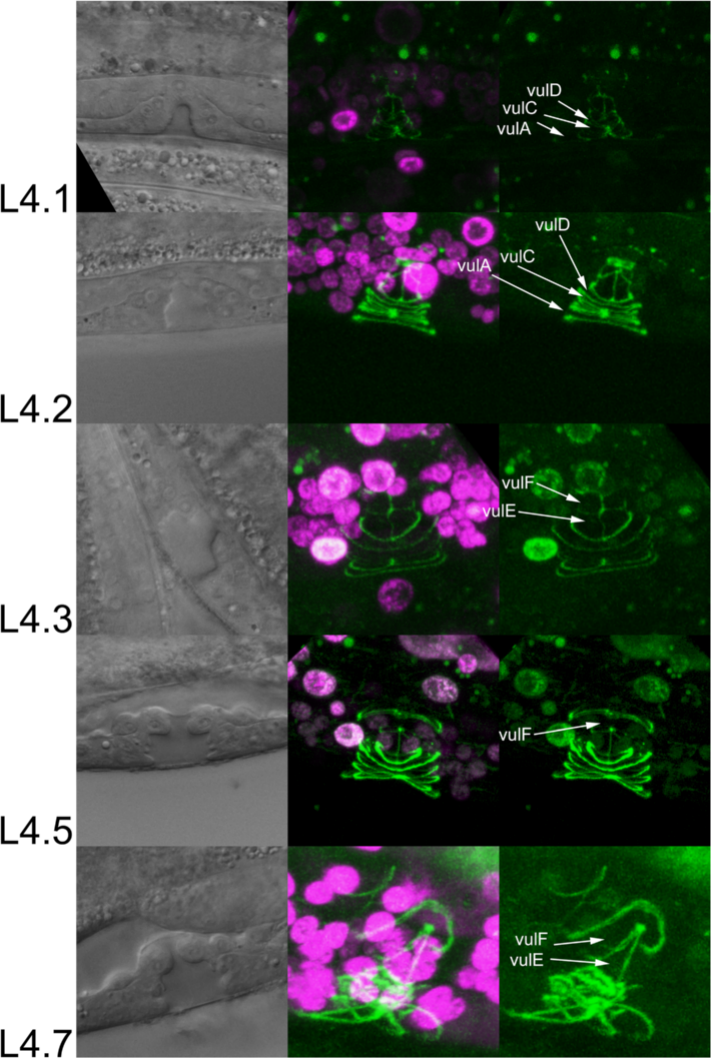
Cell fusion and sub-stages. Cell-cell junctions are labeled by *ajm-1::gfp* (green) whereas nuclei are labeled with *TdTomato::H2S* (magenta)*.* For each stage, Nomarski (left) and confocal (right) images are from the same worm, but taken separately on different microscopes. Junctions between syncytial hypodermis, vulval cells and the uterus are visible as rings, here viewed from the side. Prior to fusion of anterior and posterior vulval cells, the boundary between these cells are visible as vertical (dorsal/ventral) line of *ajm-1::gfp* fluorescence. In the L4.1 animal shown, vulC and vulD cells have formed toroids but have not fused. Anterior and posterior vulA cells are still migrating but have not contacted each other. In the L4.2 animal, vulA, vulC and vulD cells have fused, indicated by the absence of *ajm-1::gfp* fluorescence between anterior and posterior cells. In L4.1, L4.2 and L4.3 animals shown, vulE and vulF cells have not fused. However, in the L4.5 animal shown, the boundary between anterior and posterior vulF cells is not detectable, indicating cell fusion had taken place. In the L4.7 stage, further changes to the vulval cell shapes have occurred, but vulE cells have not fused yet

We also correlated L4 sub-stages with timing of other developmental events in the L4 stage (Table 3, Fig. 3). Following the terminal division, vulval cells undergo migration and fusion [7]. Using a strain carrying *ajm-1::gfp*, an adherens junction specific GFP reporter, we examined the state of cell migration and fusion in L4 sub-stages (Methods, Fig. 5). We found that cell migration continues from L3 into sub-stage L4.1. By the L4.2 sub-stage, in most animals examined (4 of 5), all cells had connected with their counterparts from the opposite side of the vulva. As for cell fusion, an animal examined in the L4.1 sub-stage had not fused its anterior and posterior cells (n = 1). However, in the L4.2 sub-stage, anterior/posterior fusion of vulA, vulC and vulD was observed in some animals (4 of 5), and by the L4.3 sub-stage, vulA, vulC and vulD were fused in all animals (n = 6). Therefore, cell migration is complete by the L4.2 sub-stage and cell fusions of vulA, vulC and vulD take place within one or two hours of cells becoming attached. The next cell to fuse, vulF, was unfused in the L4.2 sub-stage (n = 4), sometimes fused in the L4.3 sub-stage (3 of 6) and always fused in the L4.4 sub-stage (n = 6). (One of four L4.5 animals examined had unfused vulF cells, suggesting there may be some individual variability in the timing of cell fusion.) Finally, vulE fusion was not observed in L4.7 animals (n = 2), suggesting that this fusion takes place in L4.8 or later.

**Table 3:** Correlation of sub-stages with other developmental events in the L4

	Timing			
Stage	From L3-to-L4 molt	From hatch	Cell migration and fusion	Uterine lumen
L4.0	0.3	34.3		
L4.1	1.1	35.1		
L4.2	2.8	36.8	All cells complete migration. vulA, vulC and vulD cells fuse.	Uterine lumen has not started to form.
L4.3	4.1	38.1	vulF cells fuse.	Uterine lumen starts to form.
L4.4	5.0	39.0		Uterine lumen is fully distended.
L4.5	6.4	40.4		
L4.6	7.5	41.5		
L4.7	8.3	42.3		
L4.8	8.5	42.5	vulE cells fuse at L4.8 or later	
L4.9	8.8	42.8		
adult	9.7	43.7		

Time from hatching is inferred based on the assumption that L3-to-L4 molt takes place around 34 h after hatching

As with vulval development, the uterus undergoes a complex morphogenetic process during the L4 stage [28]. We did not attempt to correlate all known developmental events in the uterus with our sub-stages. Instead, we looked at an easily assayed aspect of uterine development, the generation of the uterine lumen. We found that L4.2 animals had no visible uterine lumen, whereas L4.4 animals had a large uterine lumen that appeared to be fully distended. Some animals in the L4.3 stage had an intermediate sized lumen, and it appears that the uterine lumen is generated and enlarges during this sub-stage.

## Discussion

### Utility of morphology-based staging in studies of late vulval development

An alternative to morphology-based staging is to carry out timed experiments. Individuals or populations at a particular developmental time (e.g. exit of the L3-to-L4 molt lethargus) can be selected, and further observations can be carried out at specific subsequent time points. The major advantage of morphology-based staging is that it is much less laborious. Using this system, any given animal in the L4 stage, provided that it has normal vulval development, can be staged with approximately one hour resolution.

The precise control of the temporal sequence of events during development is a problem that is not fully understood. Although the heterochronic pathway is well characterized, the sequence of events within each stage has received less attention. In particular, development of organs like the vulva requires a complex sequence of morphological changes within a single developmental stage, suggesting that there are additional timing cues which regulate specific developmental events at specific sub-stages. Whether these timing cues are organ-specific is unknown, as is the relationship between these sub-stage cues and the heterochronic pathway. L4 sub-stages described in this report should facilitate the analysis of morphogenetic or gene expression events which occur at specific time points within the L4 stage.

## Conclusions

Communication and reproducibility of results is enhanced by definitions of terms. WormBase has developed an ontology of nematode life stages that reconciles most of the terms used in the literature (W. Chen and P.W.S, unpublished results). The sub-stages we have defined here have been added to the Life Stage Ontology. This addition will allow researchers to annotate gene expression and other experiments with these stages, thus providing a more accurate depiction of their observations.

## Methods

### *C. elegans* culture and timing of L4 sub-stages

*C. elegans* strains were cultured using standard methods on NGM agar plates seeded with OP50 at 20 °C [29]. The N2 Bristol strain was used as the wild-type. To determine the precise timing of each developmental sub-stage, a population of N2 wild type worms were synchronized using a modified version of the egg-laying protocol described by Lionaki and Tavernarakis (2013) [30]. First, gravid hermaphrodites were allowed to lay eggs on seeded NGM plates for one hour and were subsequently removed. Next, the plates were incubated at 20 °C for approximately 40 h and screened for molting L3 animals based on pale appearance, lack of motility and absence of pharyngeal pumping. The molt-stage worms were then transferred to new seeded NGM plates. Finally, these synchronized populations were examined by Nomarski microscopy as described [25] at specific time points and the sub-stages were determined.

### GFP expression

To analyze the gene expression pattern in various L4 sub-stages, semi-synchronized populations of GFP reporter strains were generated by synchronized egg-laying as described above. After 40 to 50 h at 20 °C, L4 animals were picked randomly and analyzed for fluorescence and the sub-stage.

Strains used are: NH2466 *ayIs4[egl-17::gfp]*; PS3504 *syIs54[ceh-2::gfp]*; PS3475 *syIs51[cdh-3::gfp]*; JU486 *mfIs4[egl-17::yfp; daf-6::cfp]*; PS3664 *syIs65*[*B0034.1::pes-10::gfp]*; PS3527 *syIs61[F48B8.6::gfp]*; MH1564 *kuIs36[egl-26::gfp]*; OP171 *wgIs171[egl-38::TY1::EGFP::3xFLAG]*; BC15833 *sEx15833[best-13::gfp]*; ZF1638 *qwEx197[pax-2::yfp]*; ZF1639 *qwEx198[pax-2::yfp]* [4, 31–33].

Of the transgenic strains we examined, two (PS3664 *syIs65*[*B0034.1::pes-10::gfp]* and PS3527 *syIs61[F48B8.6::gfp]*) were found to be expressed only in the adult and not in L4, as reported previously [4]. The *pax-2::yfp* transgenes were generated in this study by fusing 3.1 kb sequence upstream of the *pax-2* gene to the *yfp* (yellow fluorescent protein) coding region. Although this fragment is known to contain an enhancer element active in vulval cells, our reporter failed to express YFP in the vulval tissue for unknown reasons.

To assay for cell fusion, we crossed NW1615 *plx-1(ev724) jcIs1[ajm-1::gfp]*; *him-5(e1490)* or NW1072 *smp-1(ev715); jcIs1[ajm-1::gfp]*; *him-5(e1490)* hermaphrodites with EG7959 *unc-119(ed3); him-5(e1490) oxTi405 [eft-3p::TdTomato::H2B::unc-54 3'UTR + Cbr-unc-119(+)]* males and F1 progeny carrying *oxTi405* were examined. Confocal images were obtained using Zeiss LSM700 confocal microscope. Both confocal and conventional fluorescence microscopy were used to determine whether the cells were fused.
